# Omega-3 Fatty Acids and Neuroinflammation in Depression: Targeting Damage-Associated Molecular Patterns and Neural Biomarkers

**DOI:** 10.3390/cells13211791

**Published:** 2024-10-29

**Authors:** Ikbal Andrian Malau, Jane Pei-Chen Chang, Yi-Wen Lin, Cheng-Chen Chang, Wei-Che Chiu, Kuan-Pin Su

**Affiliations:** 1 Mind-Body Interface Research Center (MBI-Lab), China Medical University Hospital, Taichung 404, Taiwan; ikbalgan@gmail.com (I.A.M.); peko80@gmail.com (J.P.-C.C.); 2 Graduate Institute of Biomedical Sciences, College of Medicine, China Medical University, Taichung 404, Taiwan; 3 Child Psychiatry Division, Department of Psychiatry, China Medical University Hospital, Taichung 404, Taiwan; 4 Graduate Institute of Acupuncture Science and Chinese Medicine Research Center, College of Medicine, China Medical University, Taichung 404, Taiwan; yiwenlin@cmu.edu.tw; 5 Department of Psychiatry, Chung Shan Medical University Hospital, Taichung 402, Taiwan; cshy1988@csh.org.tw; 6 School of Medicine, Chung Shan Medical University, Taichung 402, Taiwan; 7 Department of Psychiatry, Cathay General Hospital, Taipei 106, Taiwan; ppk11642@gmail.com; 8 School of Medicine, Fu Jen Catholic University, Taipei 242, Taiwan; 9 An-Nan Hospital, China Medical University, Tainan 709, Taiwan

**Keywords:** omega-3 polyunsaturated fatty acids, DAMPs, depression, HMGB1, S100β, NSE

## Abstract

Major Depressive Disorder (MDD) is a prevalent mental health condition with a complex pathophysiology involving neuroinflammation, neurodegeneration, and disruptions in neuronal and glial cell function. Microglia, the innate immune cells of the central nervous system, release inflammatory cytokines in response to pathological changes associated with MDD. Damage-associated molecular patterns (DAMPs) act as alarms, triggering microglial activation and subsequent inflammatory cytokine release. This review examines the cellular mechanisms underlying MDD pathophysiology, focusing on the lipid-mediated modulation of neuroinflammation. We explore the intricate roles of microglia and astrocytes in propagating inflammatory cascades and discuss how these processes affect neuronal integrity at the cellular level. Central to our analysis are three key molecules: High Mobility Group Box 1 (HMGB1) and S100 Calcium Binding Protein β (S100β) as alarmins, and Neuron-Specific Enolase (NSE) as an indicator of neuronal stress. We present evidence from in vitro and ex vivo studies demonstrating how these molecules reflect and contribute to the neuroinflammatory milieu characteristic of MDD. The review then explores the potential of omega-3 polyunsaturated fatty acids (ω-3 PUFAs) as neuroinflammation modulators, examining their effects on microglial activation, cytokine production, and neuronal resilience in cellular models of depression. We critically analyze experimental data on how ω-3 PUFA supplementation influences the expression and release of HMGB1, S100β, and NSE in neuronal and glial cultures. By integrating findings from lipidomic and cellular neurobiology, this review aims to elucidate the mechanisms by which ω-3 PUFAs may exert their antidepressant effects through modulation of neuroinflammatory markers. These insights contribute to our understanding of lipid-mediated neuroprotection in MDD and may inform the development of targeted, lipid-based therapies for both depression and neurodegenerative disorders.

## 1. Introduction

Neuroinflammation, characterized by the activation of microglia and astrocytes, has been implicated in the etiology and progression of depression [1] and neurodegenerative disorders [2,3]. This complex interplay of immune and neuronal responses contributes to neuronal damage, synaptic dysfunction, and cognitive decline [4,5]. Disruptions in microglia function could be a key factor in the development of depression [6] and neurodegenerative diseases. Individuals with a history of depression may be more susceptible to certain neurodegenerative diseases later in life, including dementia, Alzheimer’s disease (AD), and Parkinson’s disease (PD), suggesting a common underlying vulnerability in brain function [7,8,9]. Microglial dysfunction has been implicated in frontotemporal dementia (FTD) [10]. In AD, microglial cells become activated in reaction to abnormal protein accumulations, such as amyloid-β (Aβ) plaques and neurofibrillary tangles (NFTs) [11,12,13]. Similarly, in PD, the accumulation of alpha-synuclein (α-syn) protein, a key feature of PD, induces neuroinflammation. Microglia are activated by α-syn aggregates, which further exacerbate the inflammatory response [14,15]. Indeed, excessive activation of microglia leads to the release of pro-inflammatory cytokines, reactive oxygen species (ROS), and other inflammatory mediators, which damage neurons and synapses [16,17]. Chronic inflammation can contribute to neuronal damage and dysfunction, leading to elevated neuronal-damage-related biomarkers. An exaggerated inflammatory response induced by pro-inflammatory cytokines can have detrimental effects on glial cell functions and result in neuronal damage within the brain [18]. Meanwhile, studies have examined markers associated with glial and neuronal damage in depression, focusing on damage-associated molecular patterns (DAMPs) such as S100 calcium binding protein β (S100β) [19,20,21], high mobility group box 1 (HMGB1) [22,23], and the neuronal damage marker neuron-specific enolase (NSE) [24,25] as potential biomarkers for treatment response and disease progression.

Omega-3 polyunsaturated fatty acids (ω-3 PUFAs) have garnered significant attention for their potential neuroprotective and anti-inflammatory effects [26]. Eicosapentaenoic acid (EPA) and docosahexaenoic acid (DHA) are the primary ω-3 PUFAs, demonstrating efficacy in modulating inflammatory responses, reducing oxidative stress, and promoting neuronal survival [27,28,29]. ω-3 PUFAs are essential fatty acids that the human body cannot produce on its own and must obtain through diet [30]. These lipids are integral components of cell membranes, contributing significantly to their structure, fluidity, and function [31]. Anti-inflammatory effects are achieved by releasing ω-3 PUFAs like DHA and EPA from cell membranes through phospholipase A2 (PLA2) [32,33]. These fatty acids are converted into bioactive compounds by lipoxygenase (LOX) and cyclooxygenase (COX) enzymes [34,35]. These bioactive compounds activate anti-inflammatory responses by binding to specific receptors and altering gene expression, thereby reducing inflammatory cytokines [36]. Studies suggest that ω-3 PUFAs, particularly DHA and EPA, can positively influence microglial function, including suppressing the production of pro-inflammatory cytokines [37,38] and enhancing phagocytosis clear debris and pathogens in the brain [39,40]. Specifically, previous in vitro studies demonstrated that ω-3 PUFAs are considered to have an impact on microglial activation states by promoting a less reactive and more neuroprotective phenotype of microglia [41,42,43], which can contribute to better brain health and reduced neuroinflammation. In particular, the role of ω-3 PUFAs is to inhibit the activation of microglia and subsequent inflammatory responses [44]. Prior studies found that ω-3 PUFAs could interfere with the binding of HMGB1 and S100β to their receptors, resulting in reduced release of these biomarkers [44,45,46] and improvement of neuronal damage or injury in glial and neuronal cells as reflected by decreased NSE [47]. ω-3 PUFAs have been found to have beneficial effects not only for depression [48] but also for neurodegenerative diseases, including PD and AD [49]. Hence, this review explores the complex interplay between ω-3 PUFAs, neuroinflammation, and neurodegenerative diseases, with a specific focus on the role of DAMPs and neuronal damage biomarkers. By understanding the mechanisms underlying these interactions, novel therapeutic strategies can be developed to target neuroinflammation in depression.

## 2. Microglial and Astrocyte Activation and Inflammatory and Neurodegenerative Pathways in the Neurobiology of Depression

The neurobiological underpinnings of depression involve a complex interplay of various factors, including the activation of microglia [1,50,51], the resident immune cells in the brain. In individuals with depression, there is evidence suggesting that microglia become activated, triggering an inflammatory response within the brain [52]. On the other hand, astrocytes also play a crucial role in neurodegeneration, inflammation, and depression [53,54]. Upon stress, astrocytes, like microglia, become activated and stimulate the release of anti-inflammatory cytokines [55]. In contrast, the presence of pro-inflammatory cytokines, especially IL-1β, can prompt astrocytes to secrete neurotrophic factors vital for neuron survival [56]. Furthermore, hippocampal astrocytes play a role in mediating depressive behavior induced by chronic stress [57]. Activated glial cells, notably microglia and astrocytes, are key players in the inflammatory pathways linked to depression [58,59,60]. Microglia and astrocytes, the innate immune cells residing in the central nervous system (CNS), have been demonstrated to prominently generate inflammatory cytokines to uphold neurobiological homeostasis after receiving stimuli [61,62]. This activation is believed to contribute to alterations in neuroplasticity [63], neurotransmitter regulation [64], and the stress response system [65], all of which are implicated in the development and progression of depression. The immune cells of the brain can be activated by various danger signals or stimuli [66]. The secretion of damage-associated molecular patterns (DAMPs), such as S100β and HMGB1, plays a crucial role in response to cellular damage and stress. These DAMPs act as alarm signals, alerting the immune system to inflammation-induced depression. These alarm signals can trigger the activation and polarization of resting microglial cells. However, when microglial cells encounter DAMPs like S100β and HMGB1, they become activated and shift into a pro-inflammatory state. This polarization process leads to the release of inflammatory cytokines that facilitate communication between cells during immune responses. The elevated levels of certain cytokines are associated with inflammation in the brain, including interleukin-1 beta (IL-1β), interleukin-6 (IL-6), and tumor necrosis factor-alpha (TNF-α) [67,68,69], which has been linked to the pathophysiology of depression. Moreover, inflammation-induced depression is related to the activation of the microglia to modulate neuronal function [6,70]; thus, the dysfunction of neuron and microglia interaction is an important factor in the development of depression [1,71]. Activated microglia release cytokines and signaling molecules that may affect the structure and function of neurons, potentially leading to neuronal damage and impairing neural circuits involved in mood regulation [72]. As a result, atypical or chronic microglia activation and functioning disrupt neurogenesis in the dentate gyrus of the hippocampus, affecting the development and progression of various neurodegenerative diseases [2].

## 3. The Role of HMGB1 and S100β in Stress-Induced Inflammation, Oxidative Stress, and Neurodegeneration

**Figure 1:**
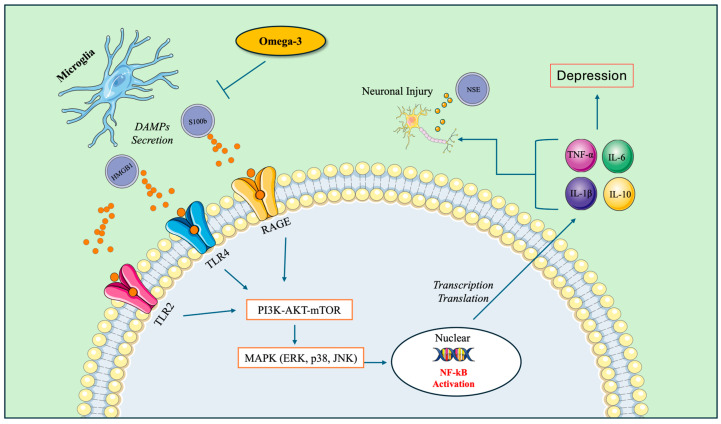
Potential pathway of modulation of ω-3 PUFAs on S100β, HMGB1, and NSE in inflammation-induced depression.

DAMPs released from damaged or stressed cells, like HMGB1 and S100 proteins, act as danger signals that activate immune cells, leading to inflammatory responses [73,74,75]. Specifically, S100 proteins are released upon cellular stress or injury [76], while HMGB1 is present in the nucleus of most cells and is actively released during cell damage, necrosis, or as a response to inflammation [77,78]. DAMPs interact with specific receptors on immune cells, such as microglia, triggering inflammatory pathways. HMGB1 or S100β interacts with pattern recognition receptors (PRRs), such as toll-like receptors (TLRs), mainly TLR2, TLR4, and the receptor for advanced glycation end product (RAGE) [73,79,80], leading to a cascade of intracellular signaling mechanisms, such as the PI3K-AKT-mTOR pathway, which is involved in cell survival, growth, and metabolism [81], and MAPK (ERK, p38, JNK), involved in regulating inflammation, stress responses, and programmed cell death [82]. In result, they can trigger intracellular signaling pathways that lead to the activation of nuclear factor-kappa B (NF-κB) [83,84,85]. Once activated, NF-κB translocates to the cell nucleus, where it promotes the transcription of pro-inflammatory genes, including cytokines, chemokines, and other mediators involved in inflammation [86]. The overproduction of inflammatory cytokines damages neurons, resulting in neuronal damage or injury [87], leading to neurogenerative diseases [88]. Figure 1 shows the details of pathway of stress-induced neuroinflammation leading to depression.

In an ex vivo study, stress was observed to induce the upregulation of the RAGE. The silencing of HMGB1 in the rostral ventrolateral medulla (RVLM) results in the reduction of RAGE protein expression [89]. This suggests that HMGB1 is a key mediator in the pathway through which stress induces RAGE upregulation. On the other hand, an in vitro study with cell lines found that silencing S100β protected cells, reducing cell death and significantly lowering oxygen radical and nitric oxide synthase activity [90]. The study shows that silencing S100β in cell lines protects cells from stress-induced damage by reducing cell death and lowering the activity of oxygen radicals and nitric oxide synthase. These findings underscore the role of S100β and HMGB1 in mediating oxidative stress and inflammation.

## 4. Microglial Activation and Inflammation-Induced Neuronal Damage in Depression

Additionally, there is clear evidence that inflammation induced by depression is associated with elevated levels of inflammatory cytokines, which are found to be higher compared to persons without the condition [91,92,93]. Excessive activation of microglia and the release of inflammatory mediators can indeed have detrimental effects on neurons, potentially leading to neuronal injury or damage [2]. Moreover, activated microglia can both directly and indirectly interact with neurons [94]. Directly, microglia can interact with neurons through physical contact [95]. They extend their processes toward neurons, forming connections and engaging in bidirectional communication [72,96]. These interactions can involve the release of signaling molecules, such as cytokines and chemokines, which can affect neuronal function and survival [94,97]. Microglia can indirectly influence neurons by modulating the brain’s microenvironment. When activated, microglia cells release various molecules that can impact neighboring cells, including neurons [98]. For instance, they can release inflammatory cytokines or growth factors that affect neuronal activity, synaptic plasticity, and neurogenesis [94,99]. The secretion of cytotoxic molecules including inflammatory cytokines results in neuronal damage [100], as shown in Figure 1. Moreover, chronic inflammation can directly harm neurons by inducing oxidative stress, disrupting cellular signaling pathways, and promoting excitotoxicity [101]. Several biomarkers are studied in the context of neuronal damage or injury, including S100β and NSE. Though astrocytes are the main source of S100β in the brain, other cell types, including microglia and certain neural cells, might also produce amounts of S100β in neuroinflammatory conditions [102]. Following chronic inflammation, the presence of elevated NSE levels can serve as an indicator of neuronal damage or injury [103].

## 5. Lipidomic Profiling and Anti-Inflammatory Properties of Omega-3 Polyunsaturated Fatty Acids in Depression

The relationship between lipid metabolism and psychiatric disorders has been studied intensively. Inflammation-induced depression is often associated with disturbances in lipid metabolism. Lipidomic studies have consistently demonstrated that depressed persons exhibit reduced levels of omega-3 [104,105,106]. Moreover, ω-3 PUFAs have been considered for depression treatment and prevention due to their anti-inflammatory properties and perceived safety and tolerability [107,108]. ω-3 PUFAs, particularly EPA and DHA, are well-recognized for their potent anti-inflammatory properties, making them a valuable component in the management of depression. These fatty acids exert their effects by integrating into cell membranes, where they replace arachidonic acid (AA), a precursor to proinflammatory eicosanoids. This substitution reduces the availability of AA for conversion into inflammatory molecules such as prostaglandins, thromboxanes, and leukotrienes, which are synthesized via the cyclooxygenase (COX) and lipoxygenase (LOX) pathways [35]. Consequently, the overall inflammatory response is diminished. Furthermore, ω-3 PUFAs lead to the production of specialized pro-resolving mediators (SPMs), such as resolvins, protectins, and maresins [109]. These metabolites actively terminate ongoing inflammatory processes by reducing the infiltration of inflammatory cells, inhibiting the release of proinflammatory cytokines, and promoting the clearance of cellular debris. For instance, resolvin E1, derived from EPA, has been shown to suppress the activation of the NF-κB pathway, a key regulator of inflammation, thereby decreasing the production of proinflammatory cytokines [110]. Chronic inflammation, influenced by these pathways, also contributes to the progression of neurodegeneration [74]. Depressed patients frequently exhibit elevated levels of proinflammatory cytokines and reduced levels of anti-inflammatory cytokines such as IL-4, IL-1β [111], and IL-6 [112]. ω-3 PUFAs can counteract this imbalance by reducing the production of proinflammatory cytokines and enhancing anti-inflammatory signaling, which may improve depressive symptoms and prevent the progression of neuroinflammation. In addition to their effects on eicosanoids and cytokines, ω-3 PUFAs impact oxidative stress pathways, which are closely linked to inflammation. By modulating the activity of enzymes such as COX-2 [113], ω-3 PUFAs reduce the production of reactive oxygen species (ROS) and nitric oxide [114], both of which contribute to inflammatory damage in tissues.

Studies have shown that individuals with more severe depression tend to have lower levels of ω-3 PUFAs [115,116,117], which correlates with increased inflammation and oxidative stress. Additionally, depressed individuals often have an imbalanced ratio of omega-6 (ω-6) to ω-3 PUFAs, which may exacerbate inflammatory responses [118]. This imbalance underscores the importance of ω-3 PUFAs in modulating inflammation and supporting mental health. Meta-analysis studies of randomized controlled trials have shown that ω-3 PUFA supplementation contributed to better improvement of depression [48,119]. Moreover, the potential of ω-3 PUFAs to mitigate inflammation suggests their therapeutic role in not only reducing depressive symptoms but also in preventing the progression of neuroinflammatory and neurodegenerative processes associated with mood disorders [26]. Emerging research has pointed to ω-3 PUFAs as mediators of inflammatory response [120], with their deficiency linked to increased oxidative stress and depressive severity [121,122]. As our understanding of these mechanisms deepens, ω-3 PUFAs may become an integral part of comprehensive treatment strategies for depression and other inflammatory conditions.

## 6. Omega-3 Polyunsaturated Fatty Acids Inhibiting Damage-Associated Molecular Pattern-Mediated Toll-like Receptors and the Receptor for Advanced Glycation End-Product Activation in Depression

Through modulation of TLR expression and function, ω-3 PUFAs can downregulate the expression of TLRs on immune cells and cause fewer TLRs to be available on the cell surface [123,124]; the binding sites for S100Β and HMGB1 are reduced, thereby decreasing the likelihood of S100Β and HMGB1 interaction with its receptors. In an animal model, ω-3 PUFAs also disrupted RAGE, the receptor specifically for HMGB1 [125]. Figure 1 illustrates that upon the release of S100β and HMGB1, they predominantly bind to specific receptors such as TLRs (TLR4, TLR2) and RAGE. This binding sets off the activation of the NF-κB pathway, which kickstarts an immune response and results in the release of proinflammatory cytokines. Additionally, in the context of chronic inflammation, the expression levels of S100β, HMGB1, and NSE might reflect neuronal damage, indicating a potential association between sustained inflammation and neuronal injury. ω-3 PUFAs potentially interfere with S100β and HMGB1 release and impede the NF-κB pathway. Specifically, previous findings revealed that ω-3 PUFAs might alleviate depression-like symptoms by mitigating hippocampal neuroinflammation in mice subjected to chronic unpredictable mild stress (CUMS) through the regulation of TLR4 expression [126]. Another study reported that ω-3 PUFAs inhibit the HMGB1-mediated activation of the TLR4/NF-κB signaling pathway in a traumatic brain injury (TBI) model [44]. Moreover, both EPA and DHA equally inhibited the messenger ribonucleic acid (mRNA) expression of S100β in the hippocampus of ageing rats [45]. The mechanism of inhibiting the NF-κB pathway can lead to a suppression of immune system activation, which includes a reduction the in production and release of inflammatory cytokines [127]. A prior study reported that ω-3 PUFAs exhibit a decrease in inflammatory cytokines such as IL-1β, IL-6, and TNF-alpha alongside a reduction in depressive symptoms [128]. This reduction in inflammatory cytokines is often associated with the anti-inflammatory properties attributed to ω-3 PUFAs. Moreover, secretion cytokines can lead to neuronal damage under inflammation-induced depression [129,130]. NSE is an enzyme primarily found in neurons, and its expression can be directly indicative of neuronal injury or damage [131]. Furthermore, in rats in a TBI model, ω-3 PUFAs were found to decrease the release of serum levels of NSE [132]. In a clinical study involving patients with TBI who received ω-3 PUFA treatment, the serum levels of both S100β and NSE were notably reduced after 7 days when compared to the control group [47].

## 7. Effects of Omega-3 Polyunsaturated Fatty Acids in Damage-Associated Molecular Patterns and Neuronal Damage Biomarkers

ω-3 PUFAs modulate the activation profile of microglia, directing them towards an anti-inflammatory or neuroprotective phenotype [37,39]. This modulation holds the potential to attenuate neuroinflammation, restore neural functionality, and potentially impede the progression or recurrence of depression [133]. Some studies have suggested that ω-3 PUFAs, particularly the EPA and DHA found in fish oil, may have antidepressant effects [134,135,136,137]. These fatty acids are involved in brain function and have been linked to mood regulation, potentially impacting the neurotransmitter pathways related to depression [133,138]. HMGB1, S100β, and NSE are biomarkers associated with various aspects of brain function and damage. HMGB1, for instance, is mainly involved in inflammation and immune response [139], while S100β and NSE are associated with brain injury and neuronal damage [140].

While there is evidence supporting the potential role of ω-3 PUFAs in alleviating depression symptoms, studies specifically examining their effect on HMGB1, S100β, and NSE in individuals with depression are sparse. Research often focuses on clinical outcomes, such as mood improvement [141,142,143,144,145,146,147], rather than analyzing these specific biomarkers. The mechanisms underlying the potential impact of ω-3 PUFAs on these biomarkers in depression are not fully understood. ω-3 PUFAs might influence inflammation and neuroprotection, which could indirectly affect these biomarkers, but direct evidence is lacking. Given the complex nature of depression and the multifactorial aspects of both ω-3 PUFAs and the biomarkers in question, more targeted studies are necessary to explore how ω-3 PUFA supplementation might affect HMGB1, S100β, and NSE levels in individuals with depression. This study could shed light on potential mechanisms and provide insights into personalized treatment approaches for depression. There have not been studies specifically dedicated to investigating the protective effects of ω-3 PUFAs on specific biomarkers such as S100β, HMGB1, and NSE in depression across animal models as well as pre-clinical and clinical research specifically related to depression. However, some studies have shown that ω-3 PUFAs can modulate the inflammatory response following TBI, potentially reducing HMGB1 [77], S100β [47], and NSE [47,132] levels and mitigating the associated damage.

### Omega-3 Polyunsaturated Fatty Acid Effects on HMGB1, S100b, and Neuron-Specific Enolase in Prior Studies

We conducted a comprehensive literature search to evaluate the effects of ω-3 PUFAs on the biomarkers HMGB1, S100b, and NSE. The search was performed using two major databases, PubMed and Web of Science, and included studies published up until October 22, 2024. We used the keywords “HMGB1 AND Omega-3,” “S100b AND Omega-3,” and “Neuron Specific Enolase AND Omega-3” to explore the relationship between Omega-3 and these markers. The search results showed that for HMGB1 and ω-3 PUFAs, PubMed listed 25 studies, while Web of Science had eight. For S100b and ω-3 PUFAs, both databases returned seven studies. For NSE and ω-3 PUFAs, PubMed had 15 studies, while Web of Science listed six.

**Figure 2:**
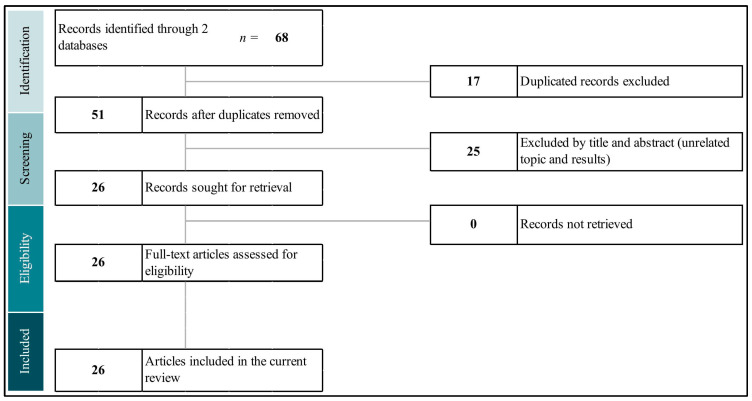
Selection process for included studies.

The detailed process of study selection is depicted in Figure 2. Our inclusion criteria focused on studies that specifically assessed the impact of ω-3 PUFAs on the levels of HMGB1, S100b, and NSE. We considered a broad spectrum of study designs, including animal models, in vitro experiments, and clinical trials across diseases. Only studies that directly measured changes in these biomarkers as a result of ω-3 PUFA administration were selected for further analysis. In contrast, studies that did not investigate the effect of ω-3 PUFAs on HMGB1, S100b, and NSE or that did not report these outcomes were excluded from our review. This systematic approach allowed us to filter out irrelevant studies and focus on those that provided relevant insights into the neuroprotective or anti-inflammatory effects of ω-3 PUFAs in relation to these HMGB1, S100b, and NSE biomarkers.

Comprehensively, studies have evaluated the impact of ω-3 PUFAs on S100β, HMGB1, and NSE in animal models and clinical contexts, as presented in Table 1. In the context of TBI, ω-3 PUFAs administered at 2 mL/kg inhibited HMGB1 acetylation and facilitated direct interactions between sirtuin 1 (SIRT1) and HMGB1 by enhancing SIRT1 activity [77], thus, reducing NF-kB activity. Similarly, ω-3 PUFAs at a dose of 0.4 g/kg/day demonstrated protective effects in the management of obstructive jaundice by reducing HMGB1 activation [148]. High doses of ω-3 PUFAs (6.0 mL/kg/day) following small bowel transplantation (SBTx) significantly reduced the expression of HMGB1 and its receptor RAGE [46]. In a study on ischemic brain damage in ovariectomized rats, EPA at 4 mg/kg downregulated HMGB1-related molecules and attenuated ischemic brain damage [149]. Studies on intestinal ischemia-reperfusion injury showed that EPA at 0.3 g/kg/day conferred protective effects by alleviating inflammation and reducing both injury severity and HMGB1 expression [150,151] and had some protective effects in relieving inflammation by inhibiting the expression and signal transmission of TLR4 mRNA [151]. An in vitro study demonstrated that 200 μM DHA induced the translocation of HMGB1 to the cytoplasm in breast cancer cells [152]. A pilot randomized controlled trial (RCT) in older adults (65–85 years) indicated a significant lowered level of HMGB1 in the ω-3-enriched group [153]. Moreover, in a study investigating aging-associated cognitive decline, EPA and DHA at doses of 500 mg/kg/day were found to equally inhibit the mRNA expression of S100β in the hippocampus of aging rats [45]. For the NSE biomarker, a study on TBI revealed that serum NSE activity was significantly lower in rats supplemented with 300 mg/kg of ω-3 PUFAs compared to untreated controls [132]. Lastly, clinical studies on severe TBI patients showed that by day 7, those treated with ω-3 PUFAs had significantly lower levels of NSE and S100β compared to the control group [47] but no significant effect of ω-3 PUFAs on S100β in septic patients [154]. Most of the included studies highlighted a positive impact, showing a decrease or suppression in the expressions of these three biomarkers. However, there is no specific study focused on assessing the effect of ω-3 PUFAs on S100β, HMGB1, and NSE in depression.

Across numerous animal models and in vitro studies, ω-3 PUFAs and RvD1 (Resolvin D1), a specific pro-resolving mediator that is synthesized from ω-3 PUFAs, particularly EPA, consistently demonstrate a significant reduction in HMGB1 [44,46,125,155,158,159,161,163,164,165], S100β [160], and NSE [156,162] levels in response to diverse conditions like TBI, ischemia-reperfusion injury, cognitive decline, and inflammatory diseases. This suggests ω-3 PUFAs possess neuroprotective and anti-inflammatory properties that could play a therapeutic role in reducing neuronal and glial injury. Many studies point to the mechanism by which ω-3 PUFAs exert their effects. For HMGB1, ω-3 PUFAs often inhibit nuclear translocation and acetylation, reduce its interaction with inflammatory pathways like TLR4, NF-κB, and RAGE, and enhance SIRT1 activity. These mechanisms highlight potential ω-3 PUFAs to modulate inflammation and cellular damage across various tissues. S100β and NSE, which are key markers for glial and neuronal injury, were shown to decrease significantly in ω-3 PUFA-treated groups compared to untreated controls in studies focused on TBI and other neurological conditions. This is particularly notable in clinical studies, such as the reduction in NSE and S100β levels in patients with severe TBI, suggesting a possible therapeutic role for ω-3 PUFAs in neuroprotection. On the other hand, not all studies present conclusive positive effects. For example, one study on septic patients did not find ω-3 PUFAs to significantly affect S100β levels, indicating potential condition-specific efficacy or variable responses depending on patient characteristics, treatment protocols, or the complexity of the underlying disease. Most of the evidence is based on animal models, with relatively few clinical trials. Among the clinical studies, one pilot RCT in older adults found that ω-3 supplementation significantly improved HMGB1 levels, which is encouraging but insufficient to generalize to broader clinical settings. There is a clear gap in robust, large-scale human clinical trials that directly examine the effect of ω-3 PUFAs on these biomarkers in conditions like MDD or neurodegenerative diseases. More clinical trials are necessary to validate these findings in humans, particularly in chronic and complex conditions like MDD and other neurodegenerative diseases. We acknowledge some limitations of the study. This study is largely based on previously published data rather than original research or direct experimentation. It does not include clinical trials that specifically investigate the connection between ω-3 PUFAs and changes in key biomarkers associated with MDD. As a result, the findings are more reflective of existing knowledge in the field and do not provide new, empirical evidence to definitively link ω-3 PUFAs to alterations in biomarkers like HMGB1, S100β, or NSE in MDD patients.

## 8. Conclusions

In summary, this review explores the relationship between neuroinflammation, neuronal markers, and the potential therapeutic role of ω-3 PUFAs in depression. It highlights the significance of DAMPs and neuronal damage biomarkers as potential indicators of the progression of depression and underscores the need for a deeper understanding of these molecular mechanisms. The exploration of omega-3 PUFAs as a promising avenue for preventing neuroinflammation in depression emphasizes their potential as therapeutic interventions. Additionally, previous research has suggested that biomarkers like S100β, HMGB1, and NSE may not only reflect the progression of depression but also indicate an increased risk of neurodegenerative diseases associated with chronic depression. This underscores the importance of further investigation, particularly through clinical trials, to explore the connections between depression, DAMP biomarkers, and the anti-depressant-like effects of ω-3 PUFAs.

## Figures and Tables

**Table 1:** Omega-3 PUFAs and S100β, HMGB1, and NSE across diseases.

No.	Study Design	Marker	Treatment	Condition	Findings	Study
1	Animal Study	S100β ^⇓^	EPA (500 mg/kg/day), DHA (500 mg/kg/day)	Ageing-Associated Cognitive Decline	EPA and DHA equally inhibited the mRNA expression of S100β in the hippocampus of ageing rats.	[45]
2	Animal Study	HMGB1 ^⇓^	ω-3 PUFAs 2 mL/kg	TBI	ω-3 PUFAs inhibited HMGB1 acetylation and induced direct interactions between SIRT1 and HMGB1 by increasing SIRT1 activity following TBI.	[77]
3	Animal Study	HMGB1 ^⇓^	ω-3 PUFAs 0.4 g/kg/day	Obstructive Jaundice	ω-3 PUFA has protective effect in the management of obstructive jaundice and reduces the activation of HMGB1.	[148]
4	Animal Study	NSE ^⇓^	ω-3 PUFAs 300 mg/kg	TBI	Serum NSE activity significantly lower in rats supplemented with ω-3 PUFAs compared with TBI group (untreated).	[132]
5	Animal Study	HMGB1 ^⇓^	ω-3 PUFAs 6.0 mL/kg/day	SBTx	High levels of ω-3 PUFAs following SBTx significantly reduced the HMGB1 and RAGE expression.	[46]
6	Animal Study	HMGB1 ^⇓^	EPA 4 mg/kg	Ischemic Brain Damage in Ovariectomized Rats	EPA downregulated HMGB1 signal-related molecules and attenuated ischemic brain damage.	[149]
7	Animal Study	HMGB1 ^⇓^	EPA 0.3/kg/day	Intestinal Ischemia-reperfusion Injury	The intervention of ω-3 PUFAs reduced levels of HMGB1 and had some protective effect relieving inflammation by inhibiting the expression and signal transmission of TLR4 mRNA.	[150]
8	Animal Study	HMGB1 ^⇓^	EPA 0.3/kg/day	Intestinal Ischemia-reperfusion Injury	The injury degree and HMGB1 expression were decreased in the ω-3 PUFA group.	[151]
9	In vitro	HMGB1 ^⇓^	200μM DHA	TNBC	DHA induced HMGB1 translocation towards the cytoplasm in breast cancer cells.	[152]
10	Pilot RCT	HMGB1 ^⇓^	749 mg EPA and 397 mg DHA	Inflammation in older adults (65–85 years)	HMGB-1 improved significantly in the ω-3-enriched group.	[153]
11	Clinical Study	NSE ^⇓^ and S100β ^⇓^	ω-3 PUFAs	Severe TBI	On day 7, the ω-3 PUFA group had significantly lower expression of NSE and S100β than the control group.	[47]
12	Animal Model	HMGB1 ^⇓^	ω-3 PUFAs	Intestinal Ischemia-reperfusion Injury	Expression of HMGB1 in the PUFA group was less than control group after ω-3 PUFA treatment.	[155]
13	Clinical Study	S100β	0.12 mg/kg ω-3 PUFAs	Septic Patients	ω-3 PUFAs did not affect markers of brain injury, including S100β.	[154]
14	Animal Model	HMGB1 ^⇓^	2 mL/kg ω-3 PUFAs	TBI	ω-3 PUFAs inhibited HMGB1 nuclear translocation and secretion and decreased expression of HMGB1 in neurons and microglia.	[44]
15	Animal Model	NSE ^⇓^	0.8 g/kg ω-3 PUFAs	Pregnant Wistar Rats	NSE was reversed after ω-3 PUFA supplementation.	[156]
16	In Vitro	HMGB1 ^⇓^	RvD1	Nasopharyngeal Carcinoma Cells	RvD1 inhibited HMGB1-induced epithelial-to-mesenchymal transition.	[157]
17	In Vivo and In Vitro	HMGB1 ^⇓^	10 μM DHA	OA	DHA could attenuate the progression of obesity-related OA and exert protective effects on cartilage by inhibiting HMGB1-RAGE/TLR4 signaling pathway.	[125]
18	Animal Model	HMGB1 ^⇓^	28% ω-3 PUFA and 3% ω-6 PUFA in fish oil	Chronic Vasculopathy of Small Bowel Allografts	ω-3 PUFAs following SBTx significantly reduced the HMGB1 expression.	[46]
19	In Vitro	HMGB1 ^⇓^	6.25, 12.5, and 25 μg/mL EPA or DHA	Intestinal Porcine Epithelial Cell Injury	EPA and DHA downregulated protein expressions of HMGB1.	[158]
20	Animal Model	HMGB1 ^⇓^	300–500 ng RvD1	Ventilator-induced Lung Injury	The protective role of RvD1 is closely linked to the decreased expression of HMGB1.	[159]
21	Animal Model	S100β ^⇓^	2% of kcals from EPA + DHA	Ovariectomized Mice	ω-3 PUFA diet plus chemotherapy attenuated gene expression of S100β by downregulation.	[160]
22	Animal Model	HMGB1 ^⇓^	0.1 μg RvD1	MI	RvD1 pretreatment exhibited protective effects against MI through downregulation of HMGB1 and its related TLR4 and NF-κB expressions.	[161]
23	In Vitro and In Vivo	NSE ^⇓^	100 mg/Kg DHA	Neonatal Jaundice	ω-3 PUFAs reduce the damage caused by bilirubin, with decreased NSE.	[162]
24	Animal Model	HMGB1 ^⇓^	15 mg/kg RvD1	Ischemia-Reperfusion Injury	RvD1 attenuated IR-induced hepatocellular damage as evidenced by serum HMGB1 release.	[163]
25	Animal Study	HMGB1 ^⇓^	1 mL DHA	Hepatic Ischemia-reperfusion Injury	Expression of HMGB1 is downregulated in liver tissues after DHA supplementation.	[164]
26	Animal Model	HMGB1 ^⇓^	100 ng/kg RvD1	Post-lung Transplant Ischemia-reperfusion Injury	RvD1 signaling on alveolar macrophages attenuated HMGB1 release.	[165]

Note: ^⇓^ Decrease; NSE: Neuron-Specific Enolase; mRNA: Messenger Ribonucleic Acid; RCT: Randomized Controlled Trial; HMGB1: High Mobility Group Box 1; SIRT1: Sirtuin 1; DHA: Docosahexaenoic Acid; EPA: Eicosapentaenoic Acid; ω-3 PUFAs: Omega-3 Polyunsaturated Fatty Acids; TBI: Traumatic Brain Injury; SBTx: Small Bowel Transplantation; TNBC: Triple Negative Breast Cancer; RvD1: Resolvin D1; MI: Myocardial Infarction; OA: Obesity-Related Osteoarthritis.

## Data Availability

No new data were created or analyzed in this study. Data sharing is not applicable to this article.

## Abbreviations

AD	Alzheimer’s Disease
BBB	Blood–Brain Barrier
COX	Cyclooxygenase
CSF	Cerebrospinal Fluid
CUMS	Chronic Unpredictable Mild Stress
DAMPs	Damage-Associated Molecular Pattern Molecules
DHA	Docosahexaenoic Acid
EPA	Eicosapentaenoic Acid
ERK	Extracellular Signal-Regulated Kinases
FTD	Frontotemporal Dementia
HMGB1	High Mobility Group B 1
IL-	Interleukin
JNK	Jun N-terminal Kinase
LOX	Lipoxygenase
MAPK	Mitogen-activated Protein Kinase
mRNA	Messenger Ribonucleic Acid
mTOR	The Mammalian Target of Rapamycin
NF-kB	Nuclear Factor Kappa B
NSE	Neuron-Specific Enolase
PD	Parkinson’s Disease
PI3K	Phosphoinositide 3-Kinase
PRRs	Pattern Recognition Receptors
RAGE	The Receptor for Advanced Glycation End Products
RCT	Randomized Controlled Trial
S100β	S100 Calcium Binding Protein β
SBTx	Small Bowel Transplantation
SIRT1	Sirtuin 1
TBI	Traumatic Brain Injury
TLRs	Toll-Like Receptors
TNBC	Triple Negative Breast Cancer
ω-3 PUFAs	Omega-3 Polyunsaturated Fatty Acids
RvD1	Resolvin D1
